# Trait-anxiety, depressive symptoms, family support and life satisfaction as determinants conditioning the degree of adherence of people in pre-older adults and older adults

**DOI:** 10.3389/fpubh.2024.1336020

**Published:** 2024-04-02

**Authors:** Mariola Głowacka, Anna Polak-Szabela, Zofia Sienkiewicz, Maciej Kornatowski

**Affiliations:** ^1^Collegium Medicum, The Mazovian University in Płock, Płock, Poland; ^2^Department of Geriatrics, Collegium Medicum in Bydgoszcz, Nicolaus Copernicus University in Toruń, Bydgoszcz, Poland; ^3^Department of Nursing Development, Social and Medical Sciences, Medical University of Warsaw, Warsaw, Poland

**Keywords:** older adults, healthy aging, successful aging, adherence, depression, family support, satisfaction with life

## Abstract

The objective of the study was to determine the degree of adherence to pharmacological treatment in people in pre-older adults and older adults age groups and to analyse the correlation between selected sociodemographic parameters, severity of anxiety as a trait, symptoms of depression, a sense of family support and satisfaction with life, and adherence in people over 55 years of age. The study was conducted in a group of 2,040 people (1,406 women, 634 men) aged 55 to 100 (the average age was 65.4). The following sociodemographic variables were analysed: age, gender, education. The following scales were used: State–Trait Anxiety Inventory (STAI), Beck Depression Inventory (BDI), Satisfaction With Life Scale (SWLS) and the Multidimensional Scale of Perceived Social Support (MSPSS). The Adherence in Chronic Diseases Scale (ACDS) was used to test adherence, understood as the implementation of the therapeutic plan. The results obtained in the ACDS ranged from 6 to 28 points; the median was 24 points (21–28). The multiple coefficients of determination (multiple *R*^2^ = 0.11; *p* < 0.001) indicated an explanation of approximately 11% of the value of the ACDS dependent variable. The total correlation of all variables (multiple *R*) with the ACDS general variable in the mean correlation was 0.33. Independent factors affecting adherence assessed in the ACDS were: severity of anxiety as a trait (*p* = −0.21 ± 0.03; *p* < 0.001), family support (*p* = 0.10 ± 0.04; *p* = 0.029), severity of depression symptoms (*p* = −0.08 ± 0.03; *p* = 0.005), age of respondents (*p* = 0.07 ± 0.02; *p* = 0.003) and satisfaction with life (*p* = 0.06 ± 0 0.03; *p* = 0.027). Severity of anxiety as a trait, age, severity of depressive symptoms, a sense of satisfaction with life and family support are important factors affecting adherence.

## 1 Introduction

Failure to adhere to therapeutic indications by patients results in the risk of not improving health results and significantly increases the cost of treatment. The effectiveness of therapy depends on the regular use of medications strictly according to medical recommendations (1–4). The quality of cooperation between the patient and the physician is referred to as adherence. This term indicates the extent to which the patient’s behavior regarding medication, lifestyle changes or therapy attendance is in accordance with the directives given by their physician (5). The WHO report shows that in the process of treating patients with chronic diseases, every second patient discontinues therapy, and every fifth does not fill the prescriptions at all (6). This contributes to the loss of control over the course of the disease, deterioration of health and quality of life, increase in mortality and the number of rehospitalizations. Therefore, one of the main challenges of modern medicine is to improve therapeutic adherence (7, 8). There is ample evidence that non-compliance with medical recommendations is influenced by many factors (9, 10). The multidimensionality and complexity of adherence generates difficulties in accurately determining its predictors. Moreover, individual factors overlap and affect patients to varying degrees. The concept of adherence is a dynamic phenomenon and requires determining the factors regulating it at each stage of treatment (11). According to the World Health Organization (WHO), factors determining adherence can be classified into 5 groups: socio-economic factors, factors related to treatment, factors related to the patient, factors related to the health care system and factors related to the disease (6, 9). One of the concepts of adherence divides the factors influencing compliance with medical recommendations into intentional and unintentional. Intentional factors result from a patient’s conscious decision to disregard medical advice, driven by, for example, negative beliefs about a particular therapy, or false information about the disease. Unintentional factors include barriers that are independent of the patient’s will (cognitive dysfunctions, functional disability). In this model, a patient’s low level of adherence can be a result of the interaction of both types of factors (12, 13). Due to multiple morbidities in geriatric patients, the implementation of treatment regimens is particularly difficult in this population (14). Specifying the determinants of adherence in pre-older adults and older adults will allow for more effective and individualized interventions aimed at adherence to therapeutic recommendations.

The objective of the study was to present the correlation between the severity of anxiety as a trait, symptoms of depression, a sense of family support and satisfaction with life, and the degree of adherence, as well as to identify factors that affect the co-responsibility of patients in the therapeutic process in people over 55 years of age.

## 2 Materials and methods

### 2.1 Participants and study design

The study was carried out as part of the grant “Adherence as co-responsibility of people at pre- and senior age in the therapeutic process,” in the period from January to November 2022. Recruitment of study participants was carried out in three stages. The inclusion criteria for the study were: age ≥ 55 years; residence or registration in the city of Płock and no cognitive impairment (assessed using the MMSE scale). In the first stage, consent to participate in the study was obtained from 2,253 people. Participants were recruited from among people attending classes at the University of the Third Age and medical entities (primary health care offices), in the city of Płock—in the Masovian Voivodship, approx. 115 km from the capital of the country—Warsaw. Płock is a city with 113,660 inhabitants (as of December 31, 2022). In the second stage, the MMSE (Mini Mental State Examination) was performed for all participants. People who did not show cognitive impairments, i.e., obtained 27–30 points in the MMSE, were qualified for the third (proper) stage of the study. In total, 2,102 people (93%) were qualified. Participants chose the method of completing the questionnaire—in paper form to be completed at the recruitment site or at home, or electronic form. The electronic questionnaire was prepared and con-ducted on the dedicated LimeSurvey platform (LimeSurvey GmbH, Hamburg, Germany). Finally, the completeness of the questionnaires was analysed, during which incomplete forms were rejected. Only complete questionnaires—2,040 (97%) were selected for analysis. In this group there were 1,406 women (68.9%) and 634 men (31.1%), aged 55 to 100. The average age was 65.4. The research material was collected using the diagnostic survey method and the clinimetric method, i.e., using standardized questionnaires.

### 2.2 Measures

The results obtained in the adherence scale were related to sociodemographic variables and to the results of questionnaires measuring selected aspects of the respondents’ emotional and social functioning. The following sociodemographic variables were analysed: age, gender, education. The following scales were used to measure aspects related to emotional and social functioning:

State–Trait Anxiety Inventory (STAI) is an adaptation of an American questionnaire. The authors of the Polish adaptation are: C. D. Spielberger, J. Strelau, M. Tysarczyk and K. Wrześniewski. This method makes it possible to determine severity of anxiety understood as a permanent internal trait and as a state appearing in response to specific external stimuli. Anxiety as a trait (Anxiety-Trait) is defined by Spielberger as a theoretical construct, meaning an acquired behavioral disposition that makes an individual susceptible to perceiving a broad range of objectively harmless situations as threatening, and reacting to them with anxiety, disproportionately strong in relation to the scope of the objective threat. Anxiety as a trait is a disposition of an anxious way of reacting. Raw results range from 20 (low anxiety) to 80 (high anxiety). The obtained raw result on the scale refers to sten norms (15).

Beck Depression Inventory (BDI) is used to measure the severity of symptoms of depression. It contains 21 questions rated according to the severity of symptoms: 0–3 points. The respondent chooses one answer which, in his/her opinion, best describes his/her condition in the indicated period (the last 7 days were assumed in the study). The severity of depression is calculated by summing the number of points from 21 questions. The following scoring is adopted: up to 11 points—no depression, 12–19 points—mild depression, 20–25 points—average (moderate) depression, 26 and more points—severe depression (16).

Satisfaction With Life Scale (SWLS) in the Polish adaptation by Z. Juczyński consists of five statements. The respondent assesses to what extent each of them relates to his/her life. The assessment of satisfaction with life is the result of comparing one’s own situation with the standards set by oneself. If the result of the comparison is positive, the result is a feeling of satisfaction. The result of the measurement is the general indicator of satisfaction with life. The raw result on the scale refers to sten norms (17).

Multidimensional Scale of Perceived Social Support (MSPSS) is a tool created by G. Zimet et al. The Polish adaptation was created by E. Hornowska and W. J. Paluchowski from 2004, and it takes into account the multidimensionality of perceived social support, dividing it into three basic sources: a significant other, family and friends (18, 19). The scale consists of twelve statements to which the respondent refers using a seven-point Likert scale, where 1 means “I strongly disagree” and 7 means “I strongly agree.”

Adherence in Chronic Diseases Scale (ACDS) was used to study adherence, understood as the implementation of the therapeutic plan. ACDS scale is dedicated to adults with chronic diseases. It contains seven questions, of which the first five concern behaviors that directly determine adherence (behaviors related to taking medications), while questions 6 and 7 refer to situations and views that may indirectly affect adherence (they address the doctor-patient relationship). Results range from 0 to 28 points. Obtaining a result below 21 points corresponds to low adherence, between 21 and 26 points to average adherence, and a result above 26 points indicates high adherence (20, 21).

### 2.3 Ethical considerations

The study was conducted in accordance with the Declaration of Helsinki and approved by Bioethical Committee of Masovian University in Płock (statute no. KB/N/BN/P/1.2021). The participants provided their written informed consent to participate in this study.

### 2.4 Statistical analysis

In the statistical analysis, parametric tests were used to compare the obtained results of the ACDS. Quantitative variables are presented as an arithmetic mean with standard deviation and confidence interval as well as median and quartile ranges. Independent samples t-test (against groups) was used to compare the medians of two data series. To assess the correlation between quantitative variables, the Pearson correlation was used. Multivariate linear regressions analysis was performed using multiple regression. Several models were constructed and the model with the highest coefficient of determination (*R*^2^) was selected. The outcome variable was an ordered qualitative variable. All independent variables were included in the model. *p* < 0.05 values were considered statistically significant. All calculations were made using Statistica 10.0 (Stat Soft Polska) and a Microsoft Excel spreadsheet using standard functions of this program and the PQStat program.

## 3 Results

### 3.1 Characteristics of the study group

There were 1,406 women (64.8 ± 8.33) and 643 men (66.8 ± 8.00) aged 55 to 100 in the study population. The average age of the respondents was 65.4 years. The standard deviation was over 12% of the mean value, which indicates an insignificant age difference. The largest group consisted of respondents aged 60 to 75—1,073 people (52.6%), the smallest group consisted of people over 90–24 people (1.2%). The group was dominated by respondents with secondary/post-secondary education—812 people (39.8%) and basic vocational education—567 people (27.8%); a smaller group of people had higher education—486 people (23.8%) and the smallest group had primary education—175 people (8.6%).

The respondents suffered from chronic diseases [hypertension—944 people (46.3%), diseases of the osteoarticular system—939 people (46.0%), vision disorders—897 people (44.0%), urinary system diseases—628 people (30.8%), lung diseases—595 people (29.2%), strokes—80 people (3.9%), permanent balance disorders—74 people (3.6%), tuberculosis—22 people (1 0.1%), AIDS—15 people (0.7%) and venereal diseases—10 people (0.5%)].

#### 3.1.1 Depression

Analyzing the study population in terms of depression symptoms (Table 1), the largest group consisted of those with no symptoms of depression—1,414 people (69.3%), while the smallest group consisted of people with severe symptoms of depression—80 people (3.9%). There was no statistically significant difference between men and women regarding the severity of depression. The highest severity of depression was presented by respondents aged over 90. Severe depression—2 people (8.3%), moderate depression—4 people (16.7%). The lowest severity was indicated by respondents aged up to 60. Severe and moderate depression—12 people each (1.8% each).

**Table 1:** Results of depression symptoms severity in age groups.

Age	Up to 60		60–75		75–90		Above 90	
Severity	Number	%	Number	%	Number	%	Number	%
No depression	551	83.0	720	67.1	131	47.0	12	50.0
Mild depression	89	13.4	229	21.3	99	35.5	6	25.0
Moderate depression	12	1.8	80	7.5	27	9.7	4	16.7
Severe depression	12	1.8	44	4.1	22	7.9	2	8.3
Total	664	100.0	1,073	100.0	279	100.0	24	100.0

#### 3.1.2 Anxiety

The largest group consisted of respondents (Table 2) with low severity of anxiety—927 people (45.4%), the smallest with a high result—268 people (13.1%). Due to the level of significance (*p* < 0.05), there was a statistically significant difference between women and men regarding the severity of anxiety as a trait. Higher severity of anxiety as a trait was obtained by women. A higher rate of high results was obtained by 205 people (14.6%). In the group of men, it was 63 people (9.9%), respectively. The highest severity of anxiety as a trait was obtained by respondents aged 75–90, high results were obtained by 42 people (15.1%) and those aged 60–75, high results—145 people (13.5%). The lowest severity of anxiety as a trait was indicated by respondents aged over 90, high results—3 people (12.5%). The results of depression remained in a statistically significant, average correlation with the results of the severity of anxiety as a trait (*r* = 0.453; *p* < 0.05).

**Table 2:** Results of severity of anxiety as a trait in age groups.

Age	Up to 60		60–75		75–90		Above 90	
STAI results	Number	%	Number	%	Number	%	Number	%
Low	348	52.4	459	42.8	107	38.4	13	54.2
Average	238	35.8	469	43.7	130	46.6	8	33.3
High	78	11.7	145	13.5	42	15.1	3	12.5
Total	664	100.0	1,073	100.0	279	100.0	24	100.0

The highest severity of anxiety as a trait was noted in the group with severe depression (Table 3). High results—40 people (50.0%) and moderate depression—49 people (39.8%). The lowest severity was indicated by respondents without depression, high results—79 people (5.6%).

**Table 3:** Results of severity of anxiety as a trait in groups with symptoms of depression measured with the BDI.

Depression	No depression		Mild depression		Moderate depression		Severe depression	
STAI results	Number	%	Number	%	Number	%	Number	%
Low	840	59.4	69	16.3	9	7.3	9	11.3
Average	495	35.0	254	60.0	65	52.8	31	38.8
High	79	5.6	100	23.6	49	39.8	40	50.0
Total	1,414	100.0	423	100.0	123	100.0	80	100.0

#### 3.1.3 Satisfaction with life

The largest number of respondents obtained high results in satisfaction with life—1,272 people (62.4%), followed by average results—469 (23.0%); the fewest respondents presented low results—299 people (14.7%).

#### 3.1.4 Social support

The mean on the MSPSS was 68.5 points—78.5% of the possible points (Table 4). The standard deviation accounted for over 18% of the mean value, which indicates an insignificant differentiation of the results. The minimum result was 12.0 points, the maximum result was 84.0 points. Support from a significant other was rated the highest—24.1 points (83.8%), support from friends—20.68 points (69.5%) was rated the lowest.

**Table 4:** Average results in the MSPSS in the study group.

Support scale	*N*	Mean	SD	Confidence −95.0%	Confidence +95.0%	Min	Max	Q25	Median	Q75
Family	2,040	23.72	4.467	23.52	23.91	4.0	28.0	23.0	24.0	27.0
Friends	2,040	20.68	5.656	20.43	20.92	4.0	28.0	16.0	21.0	24.0
Significant others	2,040	24.10	4.679	23.90	24.31	4.0	28.0	24.0	25.0	28.0
Total	2,040	68.50	12.922	67.93	69.06	12.0	84.0	64.0	70.0	77.0

#### 3.1.5 Adherence

The largest group consisted of respondents with medium adherence results—1,149 people (56.3%); the smallest group with low results consisted of 298 people (14.6%). Due to the level of significance (*p* > 0.05), there was no statistically significant difference between women and men regarding the results on the adherence scale (Table 5). The highest mean adherence was recorded in the age group 75 to 90—24.12 points and 60 to 75—24.03 points. The lowest mean adherence in the age group over 90 was 21.21 points. In terms of direct behavior, the highest mean was recorded for the age group 60–75 and 75–90, the lowest at the age over 90. In terms of intermediate situations and views, the highest mean was recorded for the age group 75–90 and 60–75, the lowest at the age over 90.

**Table 5:** Mean results of adherence in age groups.

Adherence	Direct behavior			Intermediate situations and views			ACDS		
Education	Mean	SD	Me	Mean	SD	Me	Mean	SD	Me
Up to 60	17.20	3.17	18.00	6.31	1.63	6.00	23.52	4.33	24.00
60–75	17.68	2.61	18.00	6.34	1.49	6.00	24.03	3.63	25.00
75–90	17.62	2.83	18.00	6.51	1.52	7.00	24.12	3.86	25.00
above 90	15.63	5.11	16.50	5.58	2.60	6.00	21.21	7.30	22.50

### 3.2 Analysis of the results

#### 3.2.1 Univariate analysis

To isolate factors that are statistically significant for adherence, a univariate analysis was performed, the results of which are presented in Table 6. Five significant predictors were noted: severity of anxiety as a trait, age, severity of depression, sense of satisfaction with life and family support.

**Table 6:** Univariate tests of significance for the ACDS variable (sigma-constrained parameterization; decomposition of effective hypotheses).

Effect	SS	df	MS	*F*	*p*
Absolute term	35.133	1	35.133	94.567	0.000
Age	3.223	1	3.223	8.674	0.003
Gender	0.000	1	0.000	0.001	0.980
Education	0.075	1	0.075	0.201	0.654
Severity of depression	2.939	1	2.939	7.912	0.005
Severity of anxiety as a trait	22.080	1	22.080	59.431	0.000
Sense of satisfaction with life	1.819	1	1.819	4.896	0.027
Family	1.784	1	1.784	4.803	0.029
Friends	0.675	1	0.675	1.817	0.178
Significant others	0.110	1	0.110	0.296	0.587
Error	754.179	2030	0.372		

#### 3.2.2 Multivariate analysis

Multivariate analysis was performed using multiple regression (Tables 7, 8). A multiple coefficient of determination (multiple *R*^2^) indicates an explanation of approximately 11% of the value of the dependent variable (ACDS). The entire model turned out to be statistically significant. Cumulative correlation of all variables (multiple *R*), with ACDS general variable in average correlation (0.333). Severity of anxiety as a trait, family support, age, severity of depression and a sense of life satisfaction had the greatest impact on the ACDS result in a statistically significant manner.

**Table 7:** SS test for full model against SS for residuals (sum).

Dependent variable	Multiple *R*	Multiple *R*^2^	Adjusted *R*^2^	SS Model	df Model	MS Model	SS Residual	df Residual	MS Residual	*F*	*p*
ACDS	0.333	0.111	0.107	94.162	9	10.462	754.179	2030	0.372	28.161	0.000

**Table 8:** Result of multivariate regression analysis with estimation of parameters for predicting adherence.

	Coefficient *b*	Error *b*	−95% CI	+95% CI	Standard *b*	Standard error *b*	*p* value
Absolute term	1.645	0.169	1.313	1.976			<0.001
Age	0.005	0.002	0.002	0.009	0.067	0.023	0.003
Gender	−0.001	0.030	−0.060	0.058	−0.001	0.022	0.980
Education	0.007	0.016	−0.024	0.038	0.010	0.022	0.654
Severity of depression	−0.006	0.002	−0.010	−0.002	−0.079	0.028	0.005
Severity of anxiety as a trait	−0.015	0.002	−0.019	−0.011	−0.207	0.027	<0.001
Sense of satisfaction with life	0.006	0.003	0.001	0.011	0.056	0.025	0.027
Family	0.014	0.006	0.001	0.026	0.097	0.044	0.029
Friends	0.005	0.003	−0.002	0.012	0.041	0.030	0.178
Significant others	−0.003	0.006	−0.014	0.008	−0.022	0.041	0.587

## 4 Discussion

The average degree of adherence prevailed in the study population, which was noted in 56.3% of the respondents. Almost one third of the respondents (29.1%) showed a high degree of adherence to the therapeutic plan. A low degree of adherence was noted in 14.6% of the respondents. The above results refer to the self-esteem of the respondents. It would be important to examine the degree of adherence using other methods, independent of the subjective assessment of the respondents, and to make appropriate comparisons. In literature, there are reports indicating a higher percentage of people who do not adhere to medical recommendations (6).

The lowest mean results in the general result on the ACDS scale, as well as in direct behaviors related to taking medications and in intermediate situations and views de-scribing the interaction between a physician and a patient, were recorded in the group of people over 90 years of age. In old age, a decrease in functional efficiency was observed, which could significantly hinder adherence to the therapeutic plan. In addition, the number of comorbidities increasing with age, associated with a higher percentage of cognitive dysfunctions and visual and/or hearing deficits, may have contributed to non-adherence by geriatric patients (22). The highest mean adherence was recorded in the age group 75 to 90—24.12 points and 60 to 75—24.03 points. With age, the number of comorbidities increased (23). Slightly lower average results on the ACDS scale in the group of respondents up to 60 years of age could be due to the presence of fewer diseases, lower severity of individual disease symptoms, and thus less discomfort, in relation to people from older age groups. This may explain the slightly lower level of adherence recorded in the group of the youngest respondents.

In the polish study by Kosobucka et al., conducted in a group of patients hospitalized for myocardial infarction and treated with percutaneous coronary intervention (PCI), 221 patients participated, including 63 women and 158 men. The average age of the participants was 62.9 years. High adherence to medical recommendations, measured by the ACDS scale, was observed in about 25% of patients 6 months after a heart attack. Factors influencing adherence to therapeutic recommendations were age and a history of heart attack. Patients below the age of 65 achieved higher scores on the ACDS scale, which suggests better compliance with recommendations compared to older individuals. No significant impact of other factors, such as gender or education, on the ACDS score was demonstrated (20).

In the study group, there was no statistically significant difference between women and men regarding the results on the ACDS scale. In their study conducted on a group of approximately 2,194 hypertensive patients over 65 years of age, Holt et al. (24) also observed that the incidence of low adherence rates did not differ by gender. In this study, almost half of the respondents suffered from hypertension (46.3%).

Education had no impact on the degree of adherence. Consistent results were presented by Wu et al. (9, 25), although there are reports indicating a correlation between education and the degree of adherence. People with higher education were more likely to adhere to medical recommendations (26).

In the study, the largest number of people, regardless of age, presented symptoms of mild depression. This corresponds to the data on the specificity of depressive disorders in geriatric patients. This population most often suffers from subclinical and subthreshold depression with moderate or mild severity of disease symptoms (27). Multiple regression analysis showed that the severity of depression is an independent factor affecting the degree of adherence. The greater the severity of depression, the lower the adherence to the therapeutic plan. In a meta-analysis on the impact of anxiety and depression on adherence, DiMatteo et al. (28) showed that, compared to patients without depression, there is a three times greater probability that depressed patients will not adhere to medical recommendations. The impact of depression on adherence is also described by other researchers of the subject. Depression was significantly associated with non-adherence in various diseases (29–32).

Other factors significantly influencing the results on the ACDS scale identified in the multivariate regression analysis model turned out to be family support measured by the MSPSS scale and a sense of satisfaction with life. The higher the perceived support, the higher the degree of adherence in the study group. This corresponds to research on adherence to the therapeutic plan in patients with diabetes. Numerous correlation studies have shown a positive and significant correlation between social support and adherence to recommendations concerning treatment of diabetes. Family and social support are important aspects of adherence to the principles applicable in the treatment of diabetes (33). Family support can therefore help patients implement the therapeutic plan (34, 35). Sayers et al. (36), examining patients after myocardial infarction, assessed the level of emotional and instrumental support received from relatives and its impact on adherence. They found that adherence was associated with receiving emotional support. On the other hand, Wu et al. (25) proved that the lack of family support was perceived by patients with heart failure as a factor hindering adherence to medical recommendations.

The sense of satisfaction with life and positive life balance had a positive impact on adherence to the therapeutic plan. It can be assumed that people with low satisfaction with life and negative life balance do not see the purpose of following medical recommendations and attach less importance to precise adherence to the therapeutic plan.

The predictor that had the greatest impact on adherence turned out to be the severity of anxiety as a trait measured with the STAI scale. Literature data shows that in the case of anxiety disorders, adherence, determined by the frequency of discontinuation of treatment, reaches 50% (25). The results of depression remained in a statistically significant, average correlation with the results of severity of anxiety as a trait (*r* = 0.453; *p* < 0.05). Higher severity of anxiety as a trait correlated positively with severity of depression. The coexistence of depression and anxiety as a trait negatively affected adherence to medical recommendations. The combined occurrence of symptoms of anxiety and depression has a significant impact on social functioning and behaviors focused on seeking help (37, 38). This may explain lower adherence of this group of patients to medical recommendations.

There were several recognized potential limitations affecting results of the study. The data collected was based on self-reporting, which can skew the results based on perception of what respondents think is correct behavior. It’s voluntary nature also meant there were limited amount of data points possible to collect while also retaining majority of potential respondents. The data was gathered during COVID-19 pandemic, and while there were no major lockdowns or other severely disrupting policies put in place during that period, it still might have affected the results.

Effective interventions aimed at increasing the degree of adherence in the pre- and senior population are conditioned by a compilation of important factors. Knowing the patient enables the healthcare professional to identify factors that are critical to adherence. This study highlights the importance of social support, emotional balance, and a sense of satisfaction with life to improve adherence.

## 5 Conclusion

The study found that severity of anxiety as a trait, a sense of family support, age, severity of depression symptoms and a sense of satisfaction with life are important for adherence. Severity of anxiety as a trait had the greatest impact on the result on the adherence scale. The lower the severity of anxiety as a trait, the higher the degree of adherence. Also severity of depression significantly affected adherence. A significantly higher degree of adherence was observed in people with a greater sense of satisfaction with life. People with lower family support obtained significantly lower adherence results in the study. Gender and education did not significantly affect the degree of adherence in the study group. The obtained results confirm the need for broader cooperation of all members of the therapeutic team, i.e., physicians, nurses, psychologists and pharmacologists, in educating patients on the legitimacy and effectiveness of adherence to therapeutic recommendations and monitoring the level of adherence in patients under care. Future research should focus on developing intervention strategies aimed at reducing the severity of anxiety and depression, as well as enhancing family support and life satisfaction, to improve adherence to therapeutic recommendations. Additionally, exploring methods to facilitate collaboration among various members of the therapeutic team would be valuable for optimizing patient education and monitoring adherence to medical advice.

## Data availability statement

The raw data supporting the conclusions of this article will be made available by the authors, without undue reservation.

## Ethics statement

The studies involving humans were approved by Bioethical Committee of Masovian University in Płock. The studies were conducted in accordance with the local legislation and institutional requirements. The participants provided their written informed consent to participate in this study.

## Author contributions

MG: Conceptualization, Methodology, Project administration, Validation, Writing – original draft, Writing – review & editing. AP-S: Conceptualization, Writing – review & editing. ZS: Data curation, Writing – original draft. MK: Formal analysis, Methodology, Writing – review & editing.
